# Rectal Stenosis: A Rare Anorectal Malformation

**Published:** 2012-10-01

**Authors:** Lubna Ijaz, Bilal Mirza

**Affiliations:** Department of Pediatric Surgery, The Children's Hospital and the Institute of Child Health Lahore, Pakistan

A 2-day-old male baby presented with abdominal distension and failure to pass meconium. The perineal examination revealed normally placed patent anus. A rectal examination with thermometer revealed a resistance at 2-3cm from the anal verge. A contrast enema was given to document the suspicion of rectal atresia. The contrast enema revealed a 2cm long stenosis of the rectum opening abruptly into hugely dilated proximal colon (Fig. 1). A sigmoid loop colostomy performed over a skin bridge. The patient was then discharged after 2 days with a plan of posterior sagittal management of rectal stenosis. 

**Figure:**
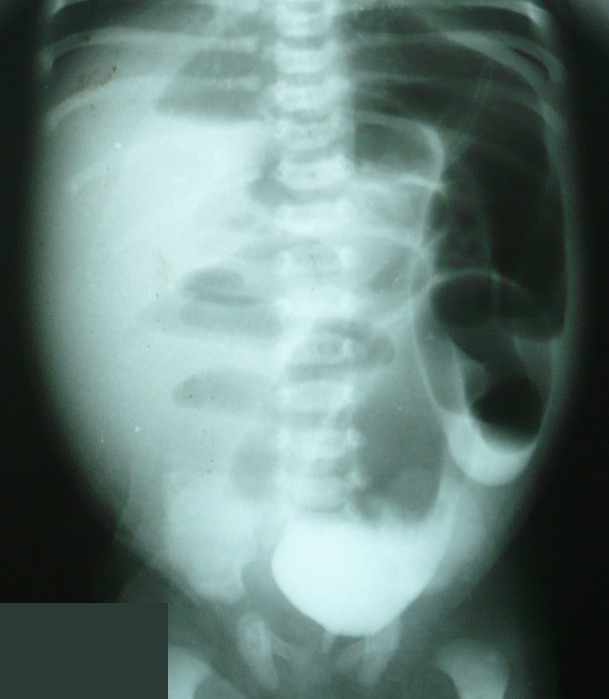
Figure 1: Contrast enema showing a 2 cm segment of rectal stenosis followed by hugely dilated colon.

## DISCUSSION

Rectal atresia and stenosis constitute about 1% of all cases of ARM with a male predominance (M:F 7:3). Various theories have been circulated as to the etiology of the malformation but most authors consider it secondary to mesenteric vascular accident [1]. The patients usually present with failure to pass meconium or pass very scantly, along with abdominal distension. On examination the anus and perineum are well developed with an anal canal of 1-2 cm before commencing the rectal atresia or stenosis. A rectal examination with thermometer or nelaton catheter usually shows a resistance to proceed beyond 1-2 cm of patent anal canal. The radiological confirmation of the diagnosis is established by taking radiographs (invertogram/lateral prone) with metallic bougie or red rubber catheter in the anal canal. The radiographs will show a distance between the bougie and the colonic gas depending upon the type of rectal atresia [2,3]. In our case, a contrast enema with gastrografin delineated 2cm long rectal stenosis entering into proximal dilated colon. Various authors considered it as a separate entity but majority considered it as ARM and therefore it was retained within rare malformation in Krickenbeck's classification. The index entity has been reported with imperforate anus, and rectal fistulae with urethra, vestibule, labia, and vaginal canal. That too indicates its strong relevance with the ARMs [1-4]. 

Many techniques have been reported for treating the condition ranging from direct rupture of the rectal diaphragm with metallic bougie to abdomino-perineal pull through procedures. The standard protocol is staged correction with colostomy formation in the first stage followed by rectoplasty or recto-rectal anastomosis through posterior sagittal approach. The prognosis is satisfactory with regard to the continence. This is attributed to the presence of normal sphincter complex and perineal musculature in this anomaly that are necessary for continence [1-3,5].

## Footnotes

**Source of Support:** Nil

**Conflict of Interest:** The co-author belonged to the editorial board, however the manuscript was independently processed by other editor and the co-author did not influence the decision making.
